# Why are drug-related deaths among women increasing in Scotland? A mixed-methods analysis of possible explanations

**DOI:** 10.1080/09687637.2020.1856786

**Published:** 2020-12-11

**Authors:** Emily J. Tweed, Rebekah G. Miller, Joe Schofield, Lee Barnsdale, Catriona Matheson

**Affiliations:** a MRC/CSO Social and Public Health Sciences Unit, University of Glasgow, Glasgow, UK; b School of GeoSciences, University of Edinburgh, Edinburgh, UK; c Drugs Research Network for Scotland, University of Stirling, Stirling, UK; d Public Health Scotland, Edinburgh, UK; e Faculty of Social Sciences, University of Stirling, Stirling, UK

**Keywords:** Drug use, gender, mortality

## Abstract

Drug-related deaths have increased significantly in Scotland in recent years, with a much greater percentage increase in deaths among women than among men. We undertook a mixed-methods project to identify explanations for this trend, comprising three parallel methodological strands: (i) an analysis of available routine data, including drug treatment data, death registrations, and surveys of people using needle exchanges; (ii) thematic analysis of interviews and focus groups with professional stakeholders and (iii) secondary analysis of interviews with women who use drugs. Results indicated that the observed trend is likely to reflect multiple, interacting causes. Potential contributors identified were: ageing; changing patterns of substance use; increasing prevalence of physical and mental health co-morbidities; changing relationships and parenting roles; changes to treatment services and wider health and social care provision; unintended consequences or poor implementation of recovery-oriented practice; and changes in the social security system. Policy responses to rising drug-related death rates require a gender-informed approach, recognising the commonalities and differences between men and women who use drugs; the diversity of experiences within each gender; and the intersections between gender and other forms of inequality, such as poverty.

## Introduction

**Figure 1:**
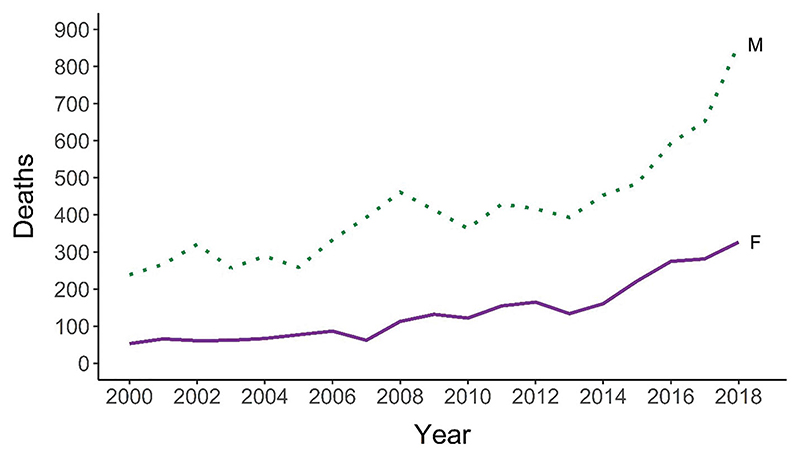
Number of drug-related deaths in Scotland 1996–2018, by gender. Source: National Records of Scotland.

Drug-related deaths (DRD) have been increasing in Scotland in recent years (National Records of Scotland, 2019b). In 2018, 1,187 deaths were registered—the highest number ever recorded and a 107% increase on 2008. Although men account for the majority of DRD, the percentage of women has increased over time, from 19% in 2004–2008 to 29% in 2014–2018. When comparing the annual averages for these two periods, the percentage increase in the number of DRDs was greater for women (212%) than for men (75%). Figure 1 shows the overall trend in number of deaths by gender.

A number of explanations have been proposed for the overall increase in DRD observed throughout the UK and a number of other high-income countries, including a prematurely ageing cohort of people who use opioids; a rebound in heroin availability in recent years; polysubstance use; physical and mental health co-morbidities; worsening socioeconomic circumstances; and changes in drug treatment provision (Advisory Council on the Misuse of Drugs, 2016). However, to date there has been limited consideration of the reasons why current trends in DRD differ by gender.

Cohort studies of people who use drugs consistently find a higher crude or absolute DRD rate among men compared to women (Mathers et al., 2013). The reasons for this observation are uncertain. While there are many commonalities between men and women who use drugs, there are several areas where experiences of substance use, harm reduction, and treatment differ in ways that may affect drug-related death risk. For instance, women appear to experience greater stigma associated with drug use, related to societal gender roles and expectations, and unique barriers to accessing treatment services (European Monitoring Centre for Drugs & Drug Addiction, 2017). There is also some evidence that women are more likely to engage in non-medical use of prescription drugs, though this may reflect greater exposure to prescription medications with the potential for abuse (Clark, 2015).

Among opioid users, men are more likely to inject and to experience episodes of imprisonment (Bird et al., 2003; European Monitoring Centre for Drugs & Drug Addiction, 2006; Shand et al., 2011), both of which are important risk factors for DRD, though studies from the National Drug Treatment Monitoring System in England have found that these factors only partly explain the observed difference in mortality risk (Pierce et al., 2016, 2018).

There is also some evidence that the impact of known risk factors varies between men and women. Increasing age is an important risk factor for DRD, but this effect appears to be especially pronounced among women (Onyeka et al., 2014; Pierce et al., 2015, 2016). There is also uncertainty about differential impacts of treatment: one cohort study from England found that women were less at risk of DRD during out-of-treatment episodes than men (Pierce et al., 2016), while others have found that, unlike deaths in which heroin only is implicated, rates of methadone-specific mortality are similar (Gao et al., 2016; Pierce et al., 2018). The extent to which these observations are due to physiological differences between the sexes versus gender inequalities in social experience remains uncertain, though direct evidence for the former appears to be limited (European Monitoring Centre for Drugs & Drug Addiction, 2017).

In light of recent trends, and evidence of important differences in the experience and vulnerability of women and men who use drugs, we undertook a mixed-methods examination of potential explanations for the rising rate of DRDs among women in Scotland in recent years.

## Methods

### Definition of DRD

In Scotland, DRDs are reported annually by National Records of Scotland (NRS) according to a standardised definition (National Records of Scotland, 2019b). In Scotland, there is a legal requirement to register deaths within 8 days, therefore there is a high level of correspondence between the year of registration of death (used by NRS for reporting) and year of death.

### Analysis of routine data

Routine national data about people accessing drug treatment services or experiencing drug-related harms were obtained from the sources described in Table 1, either from existing publications or bespoke requests. All data obtained via bespoke requests were provided in aggregate form to protect confidentiality.

### Stakeholder views

Views were sought from key professional stakeholders from statutory services, the third sector, and academia, as part of an information gathering exercise to generate and explore hypotheses for the observed trends. Stakeholders were recruited by invitations sent through existing networks including the Partnership for Action on Drugs in Scotland Harm Reduction Group (a stakeholder group convened by Scottish Government to inform drug policy) and local Alcohol and Drug Partnerships (responsible for the provision and commissioning of local alcohol and drug services in Scotland), as well as professional contacts of the authors. Potential participants were provided with an information sheet and invited to participate by email. Verbal informed consent to participate was obtained and documented at the start of the interview.

Informal semi-structured interviews (in person or by telephone) and focus groups were undertaken by RM and EJT, with fieldnotes taken during and immediately after the event. As these interviews and focus groups were recorded via field notes rather than audio recordings, no direct quotations are included in this report.

In all, we undertook 15 individual interviews; one joint interview with two participants; two focus groups; and one observed meeting of an existing community of practice. A summary of stakeholder roles is provided in Supplementary Material and an example topic guide is available on request.

### Secondary analysis of interviews with women who use drugs

To incorporate insights from women with lived experience of drug use without imposing additional research burden on them, we undertook secondary analysis of interviews conducted as part of the Older People with Drug Problems (OPDP) project. The methods of the OPDP project, which recruited women from a range of non-NHS settings across Scotland such as needle exchanges, voluntary organisations, and homelessness services, are described elsewhere (Matheson et al., 2019). Interview data were anonymized and analysis conducted by the researcher who undertook the interviews (CM).

For this project, 13 additional interviews were transcribed and used alongside 15 existing transcripts, resulting in a sample of interviews with 28 women.

### Analysis

Routine national data sources were analysed descriptively. Qualitative data was coded using directed content analysis collaboratively by RM, EJT, and CM, based on a thematic matrix reflecting potential explanations which was developed iteratively in response to emerging findings and themes. During this process, representative quotations were identified for inclusion in the paper to illustrate key themes. A copy of the thematic matrix is available on request. The results of the quantitative and qualitative analyses were combined through a narrative synthesis organised according to the key emerging hypotheses. Reflecting the mixed-methods approach of the study and its goal of developing an explanatory framework, the rest of the paper presents a findings section which integrates results and interpretation, followed by key conclusions and implications for policy and practice (Bazeley, 2015).

### Ethical approval

The project was conducted in accordance with the Scottish Government Social Research Ethics Guidance.

### Terminology

Despite the distinct meanings of the terms sex and gender, they were often used interchangeably and inconsistently in our sources. Our intention with this work was to present an account of what might contribute to the disproportionate upward trend in DRD among women, rather than undertaking a detailed analysis of the gendered nature of drug use or the relative contribution of sex and gender differences. For simplicity, we therefore use the terms women/men and gender throughout this article, though we continue to use female/male as adjectives.

## Findings

This narrative summary of the evidence examined draws on the synthesis of all methodological strands, reflecting the challenges inherent to our topic: up-to-date evidence on relevant factors in recent years is limited; what is available does not lend itself to formal hypothesis testing; and in reality the causes of this phenomenon are likely to be multiple and interacting.

Explanations are ordered by theme, rather than importance; starting with potential changes in the measurement of DRD that may have created the erroneous impression of a trend, before moving on to consider explanations relating to individual factors, communities and services, and societal trends.

### Changes in the definition and recording of DRD

Analysis of National Records of Scotland (NRS) data show that the trend in DRDs among women was not substantially affected by changes in the legal classification of drugs since 2000 (Table S2.1, Supplementary Material): the number of deaths excluded from the standard DRD figures because the substance involved was not controlled at the time of death were relatively small in number across the time series and were not higher among women. Moreover, although there are a number of artefactual factors which may affect trends in DRD rates over time and place (such as advances or changes in toxicology testing or practice), these are unlikely to differ by gender. Changes in the definition or recording of DRD are therefore unlikely to explain the trends observed.

### Changes in the population at risk

Understanding the scale of problem drug use—particularly over time—is challenging: this section therefore triangulates a number of data sources to investigate the potential contribution of changes in the population at risk. Table S2.2 (Supplementary Material) summarises evidence from community and treatment-based data sources, which suggest a largely stable gender profile among a static or falling population of people who use drugs or with drug problems in Scotland. The limited data available on duration of injecting drug use also suggest that this is unlikely to explain the differential trends by gender.

However, given the limitations of self-report or treatment data, they do not completely exclude the possibility of ‘hidden’ populations of people who use drugs (particularly prescribed drugs) which may be growing in number and/or experiencing increased risk of DRD.

Analysis of data from the National Drug-Related Deaths Database (NDRDD) found that the percentage of female decedents who were known to use drugs decreased over time from 90% in 2009 to 81% in 2016, whilst among men it has remained largely stable, at 89% and 91% respectively (Information Services Division Scotland, 2018a). In keeping with this, several professional stakeholders raised concerns about local examples of DRDs associated with prescription drugs (such as opioid painkillers and benzodiazepines) among older women not fitting the typical profile of people considered to be at risk.

In addition, the Older People with Drug Problems in Scotland (OPDP) project identified a group of people using benzodiazepines problematically, but not using opioids: women aged 35 or over were more prevalent among this group than among the overall cohort used for the project (Scottish Drugs Forum, 2017). A very high percentage of these women did not have any specialist drug treatment or drug-related hospital activity during the study period. However, mortality among those using benzodiazepines only in the OPDP project was much lower than for those with opioid use (2.3% vs 4.2%) so a hidden population of women using benzodiazepines appears unlikely to explain a higher relative increase in DRD.

In keeping with this, other stakeholders had not observed any such trend locally and the vast majority of DRDs continue to involve illicit opioids. This possibility is therefore unlikely to make a major contribution to the overall trend but may warrant further investigation.

Together the results in Table S2.2 suggest that the higher relative increase in DRDs among women compared to men is unlikely to be driven by an increase in the number of women using drugs or in their relative duration of exposure to drug-related risks.

### Ageing among women who use drugs

Routine data sources (supplementary Table S2.2) suggest that the median age of both women and men who use drugs in Scotland is increasing, with similar trends by gender over time. This is also the case for people experiencing DRD. Among people accessing IEP services and treatment, the median age is slightly higher among men, but among people who died, the reverse is true although this difference is small. These data suggest that ageing alone cannot explain the disproportionate increase in DRD among women.

**Figure 2:**
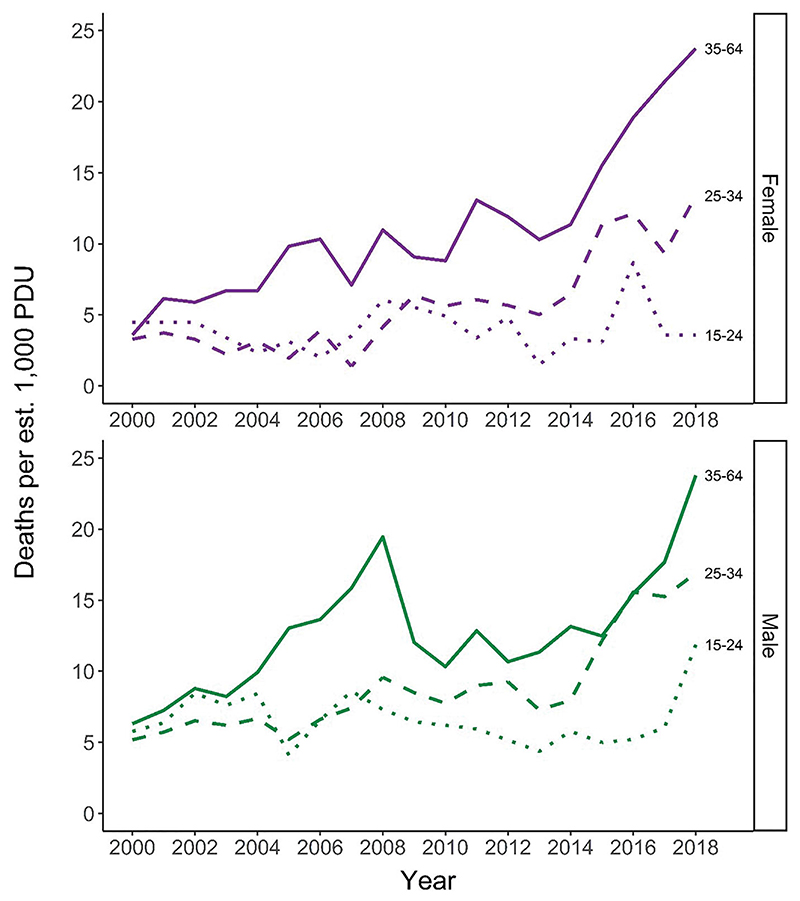
Rates of drug-related death per estimated 1000 problem drug users, by gender and age group. Source: National Records of Scotland & Public Health Scotland.

DRD rates per estimated 1000 problem drug users have increased or remained largely stable within the three age strata for which data are available (Figure 2); the picture is similar when considering DRD rates per 1000 total population, for which ten-year age strata are available (National Records of Scotland, 2019b).

It is also important to examine why age is associated with an increase in the risk of DRD, and whether this effect may vary by gender. It is unclear to what extent age exerts a direct causal effect on DRD risk (for example, through a greater burden of physical co-morbidities) or is a marker for other risk factors (such as poly-substance use, social isolation, or more complex life circumstances). Stakeholder interviews identified age as a factor that needed to be critically interrogated, arguing that it was important to understand the activities and experiences of people who use drugs as they age. For instance, as described in the subsequent section on ‘Relationships’, increasing age appears to be associated with increasing social isolation, and with greater reflections on one’s life stage and circumstances. These themes are illustrated by the quotations below from interviews with women who use drugs.

I think it’s got quite a lot to do with mental health, but sometimes I don’t know, I just, I’ve given up just now, because everybody’s been dying round about me, just giving up.

…now that I’m 35 I’m thinking “Oh my God, I’m nearly hitting 40, I’m still using gear, I haven’t got a job, a lot of mental illness, I haven’t got any kids, I’m not married. My Mum and Dad wanted more for me than that and I feel, they make me feel guilty about that you know.

Ageing effects (the result of growing older) are difficult to separate from cohort effects (the result of being born during a specific period) and period effects (the result of factors that occur at a particular time and affect all age groups equally). A recent analysis of time trends to disentangle these factors in drug-related death risk in Scotland, found that as well as ageing, a cohort effect associated with an increase in risk among those born between 1960 and 1980 was present among women but less pronounced than among men (Parkinson et al., 2018).

Age may therefore be important not only as a cause of physical vulnerability but also as a marker of wider life changes and transitions as well as historical experiences. Many of these age-related factors—whether physical, mental, or social—are potentially amenable to intervention and support.

### Changing patterns of drug use among women

**Figure 3:**
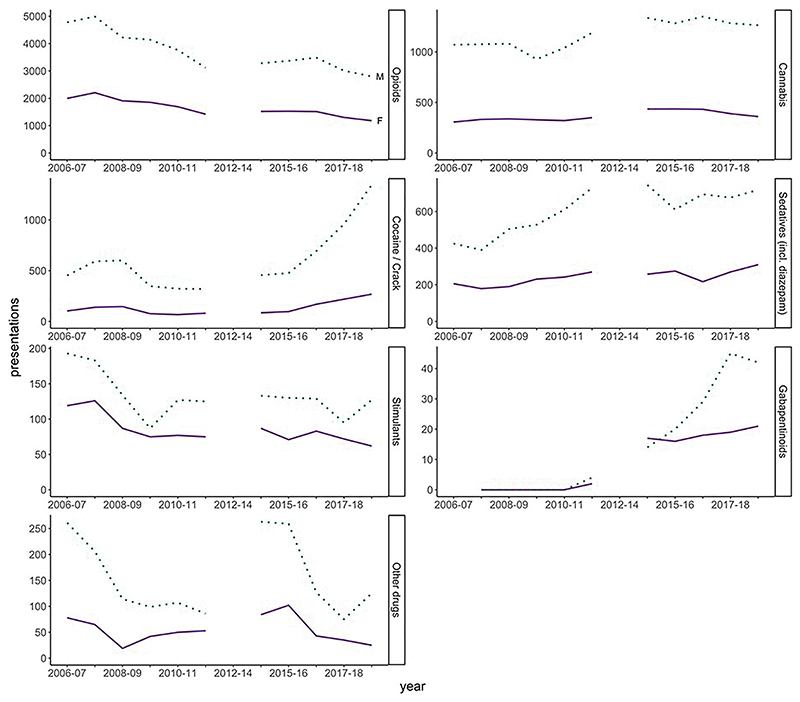
Number of individuals presenting for initial assessment in specialist drug services between 2006–2007 and 2017–2018, by financial year, gender, and main type of illicit drug. (Note different scales of *Y* axis). Note that data quality issues mean that data for 2012–2013 and 2013–2014 are not available. Source: Scottish Drug Misuse Database, Public Health Scotland.

Data sources on drug use suggest similar trends in men and women in recent years. Scottish Drug Misuse Database (SDMD) data show that opioids remain the most common drug class for which people undergoing initial assessment in specialist drug services seek help, with little difference between genders (Figure 3). With regard to other drug classes, recent years saw substantial increases in men seeking help where the main illicit drug was cocaine or gabapentinoids, outstripping smaller increases among women.

Looking at results from the Needle Exchange Surveillance Initiative (NESI) among people attending injecting equipment provision services, a similarly high percentage of both genders reported injecting heroin in the last 6 months (92% women vs 90% men). Both genders showed a substantial upwards trend in injecting cocaine and crack in recent years, though women remained less likely than men to inject these (either alone or in combination with heroin). Trends in injecting benzodiazepines and ‘legal highs’ (i.e. novel psychoactive substances) were similar by gender.

**Figure 4:**
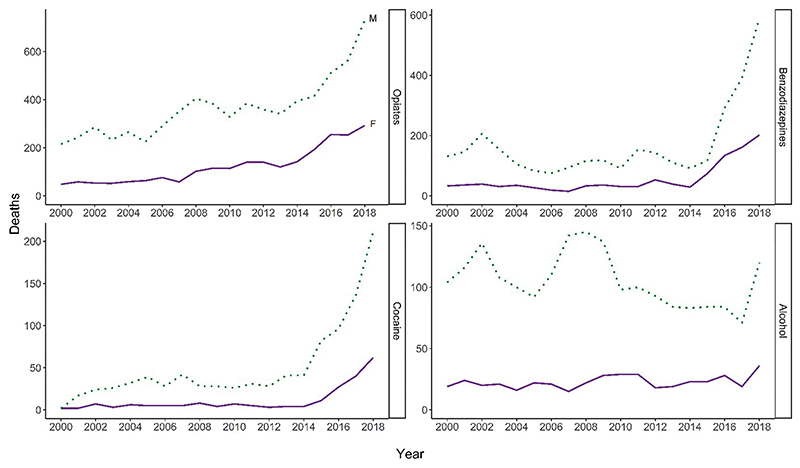
Number of deaths where opiates, benzodiazepines, cocaine, or alcohol were implicated in, or potentially contributed to, the cause of death, by gender: NRS definition*. (Note different scales of *Y* axis). Source: National Records of Scotland. NRS definition of drug-related death, based on UK Drug Strategy. These data will therefore not include deaths involving any substances uncontrolled at the time of death (e.g. an overdose of tramadol alone prior to 10 June 2014 or an overdose of etizolam prior to 31 May 2017). The dashed line delineates a change in reporting practice for drugs involved: up to 2007, some pathologists reported only those drugs which they thought directly contributed to the death, whereas from 2008, they report separately drugs which were implicated in, or which potentially contributed to the death (shown here), and those which were present, but were not considered to have contributed to the death (not shown here). Since these data record individual mentions of particular drugs, there will be multiple-counting of deaths where more than one drug is present.

Among drugs implicated in or potentially contributing to deaths, opiates remain the most common drug in both genders, though benzodiazepines and cocaine have shown rapid increases in recent years (Figure 4) (National Records of Scotland, 2019a). Trends in deaths relating to these drugs were generally more pronounced among men, except for alcohol, where the picture was similar among men and women. NRS data on decedents indicates that the percentage of deaths in which alcohol is implicated has declined among women since 2000 (data not shown), suggesting changes in alcohol consumption are unlikely to explain the observed trends. The percentage of deaths in which any opiate was implicated remained largely stable in both genders over time: among women, the percentage of deaths involving methadone has in most years been slightly higher than among men, and the percentage of deaths involving heroin/ morphine slightly lower (data not shown). This is consistent with recent findings that a higher percentage of women who inject drugs (NESI)—and women who die from DRDs (NDRDD)—receive prescribed methadone than men though this does not preclude higher rates of illicit methadone use or a greater physical susceptibility to complications of methadone treatment. The number and percentage of deaths in which methadone is implicated has increased in recent years, and declined for heroin, though both trends are similar between genders (National Records of Scotland, 2019a).

NRS data show that multiple drugs are present in the body at time of death for almost all DRD in Scotland, with the majority having multiple substances implicated (National Records of Scotland, 2019b). Although previously, the percentage of deaths in which multiple drugs (and, perhaps, alcohol) were implicated was higher among women, in more recent years trends for the two genders have converged (supplementary Figure S4.1). For most combinations, the direction of the trend over time was similar for both genders or steeper among men. The only apparent exception to this was deaths where methadone and alcohol (with or without other drugs) were implicated, where the trend appeared to be slightly steeper among women, though this was a relatively uncommon combination and total numbers were still higher among men (National Records of Scotland, 2019a).

**Figure 5:**
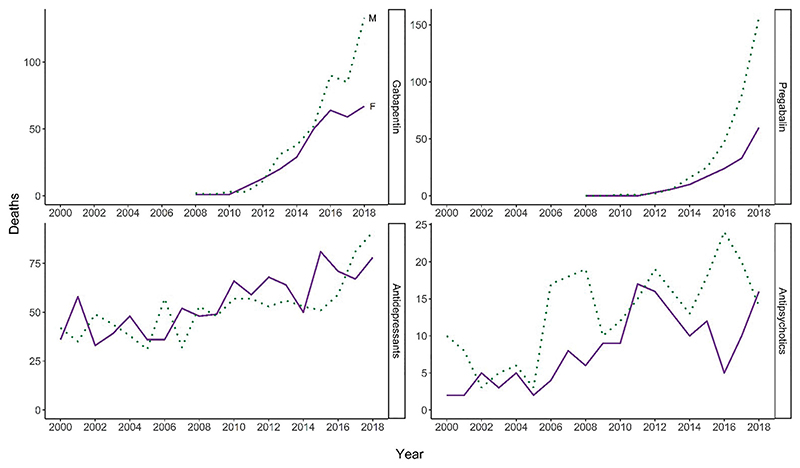
Number of drug-related deaths involving selected prescription drugs, by gender: ONS ‘wide’ definition. Source: National Records of Scotland. The ONS ‘wide’ definition includes all deaths coded to accidental poisoning, and to intentional self-poisoning by drugs, medicaments and biological substances, whether or not a drug listed under the Misuse of Drugs Act was present in the body . The dashed line delineates a change in reporting practice for drugs involved: up to 2007, some pathologists reported only those drugs which they thought directly contributed to the death, whereas from 2008, they report separately drugs which were implicated in, or which potentially contributed to the death (shown here), and those which were present, but were not considered to have contributed to the death (not shown here). More than one drug may be reported per death. These are mentions of each drug, so do not add up to the overall total.

The Office for National Statistics (ONS) ‘wide’ definition for DRD can be used to examine deaths in which prescribable drugs are implicated, since it counts all deaths from drug poisoning regardless of whether a drug listed under the UK Misuse of Drugs Act (1971) was known to be present in the body at the time of death (National Records of Scotland, 2019b). Death registration data using the ONS wide definition suggest a much smaller gender gap than for illicit drugs (Figure 5), with an especially marked rise in deaths involving gabapentinoids in both genders in recent years. Both genders have also seen gradual upward trends in the number of deaths with anti-depressants involved, with these consistently involved in a higher percentage of deaths among women than men. For anti-psychotics, although there is an apparent upward trend in recent years, the numbers are much smaller and there is no consistent gender difference.

From stakeholder interviews, prescription drugs were identified as a potential factor in the observed trend in DRDs among women. This includes both the problem use of prescription drugs by women without a history of dependence on other drugs and who do not fit the conventional profile of people at risk of drug-related harms (as discussed above under ‘Changes in the size of the population at risk’), as well as prescription drug use among women with established street drug use. Drugs identified as particular concerns include gabapentinoids and benzodiazepines, especially short-acting formulations (e.g. alprazolam). Some stakeholders identified that women were more likely to be prescribed drugs with the potential for abuse such as these, though illicit supply was also recognised as an important source. One stakeholder suggested that prescription drugs—not detected on urine screening—may be particularly appealing to women going through child protection proceedings.

Polysubstance use (of illicit or prescribed drugs, or—most commonly—both) was felt by stakeholders to be particularly problematic with increasing age, due to a loss of physiological reserve and greater prevalence of co-morbidities. It is unclear whether gender differences in harms might reflect differences in the prevalence of polydrug use or in vulnerability to its effects. Further work to investigate polysubstance use from routine data sources—such as SDMD and death registrations—is an important area for future work.

### Changes in relationships and parenting roles

The link between women having children removed from their care and risk of DRD was a recurring theme with professional stakeholders and women with lived experience. Stakeholders described how, with increasing age, women may have experienced multiple child removals, may have very limited positive family relationships, and may find the prospect of declining fertility in this context difficult. Some felt these factors were contributing to retraumatisation, hopelessness and risky consumption among women, increasing the risk of DRD. This was supported by women who use drugs’ accounts of their experiences of child protection proceedings:

Well my last time I was clean I got, I came down, got reduced and I came off it, and it wasna [wasn’t] until I got my children taken away from me last year that I went back.

I mean I think I would have stopped wi’ the methadone if there hadn’t been so many up and downs with the children getting taken off me and such, and there’s been so much, and then, I went through. My kids got taken off me, beatings from the ex, so, Women’s Aid people were involved, come up to talk to me, things like that. So there had been a lot of things that, not made me take drugs but, I chose to take them to help. But then it doesn’t help. It doesn’t seem to force away the things from your head.

On the other hand, one professional stakeholder suggested that without the risk of pregnancy and fear of child protection concerns, older women may be more able to engage with treatment services.

There are few routine data on the role of relationships and parenting in DRD. Data from NDRDD show that the percentage of women who died who were parents or parental figures increased between 2009 and 2011 and subsequently declined. However, there was no clear trend in the percentage of women who died who were living with children under 16 years at the time of death (Figure S2.2 in Supplementary Material). Among men who died, both measures have been relatively stable over time. No trend data on child protection social work activity where drugs were a factor were available for this report. This merits further investigation.

Regarding relationships more generally, increasing social isolation was identified as a growing problem by a number of stakeholders and by women themselves. Causes cited included deaths within peer groups, relationship breakdown with non-using friends and family among people who continue to use, the end of friendships with drug-using peers among people entering treatment and recovery, mental health problems, or a lack of trust in others following trauma. Some of these themes are illustrated by the quotations below from interviews with women who use drugs:

So I needed [name], because my mum wasn’t well, because obviously I’ve cut everybody out of my life that I did, anybody I knew that was all to do with drugs.

… me and my mum are so alike, I couldn’t stay with my mum, because I stayed there for a wee while, but in the end, we end up fighting, and it’s the same with my sister, my dad, he’s only like got one room, so that’s no good, and he’s not talking to me at the minute, because of my sister, so I feel like I’ve just got nobody really

These factors may be particularly salient for women, given the evidence that women’s drug use tends to be more closely linked to intimate relationships and is more highly stigmatized. Some stakeholders also highlighted a lack of gender-concordant peer support for women within existing services. On the other hand, there is some evidence to suggest that women are more able to establish new social relationships unrelated to drug use than men, and enjoy a greater level of practical and emotional support from family members (Neale, 2004).

### Experience of adversity, trauma, and violence

The only source of routine data available on this topic in Scotland is the National Drugs-Related Deaths Database, which collects information on whether the decedent was known to have been a victim of domestic violence or sexual abuse. Among those dying between 2009 and 2016, both of these experiences were much more common among women than men (overall prevalence 44% vs 5%) but there were no clear trends over time observed in either gender (Information Services Division Scotland, 2018a).

Stakeholders, however, did identify some potential factors which have changed in recent years which may interact with women’s experiences of adversity, trauma, and violence to increase the risk of drug-related harms, including death. These include:

- Changes in the social security system which make women more vulnerable within relationships. For instance, the new Universal Credit payment is claimed as a household but only paid to one individual, which may reduce women’s economic independence, as may the increased use of sanctions (Engender, 2015; Scottish Government, 2013).
- Life events or transitions more common among an ageing cohort, such as bereavements, multiple child removals, and loss of fertility, which may be traumatic in themselves and compound the effects of previous trauma.
- Changes to health and social services resulting in reduced provision (e.g. mental health), a lack of continuity (whether in services themselves or in staffing), or a less holistic approach (e.g. as a result of reduced staffing and skills within addictions services or more ‘punitive’ approaches). These changes may disproportionately affect engagement and outcomes among people with a history of trauma, who can experience particular difficulties in accessing services or establishing therapeutic relationships (Elliott et al., 2005).

Further in-depth work to explore the contributions of these factors would be beneficial.

### Co-occurring physical and mental health conditions

As described above, increasing prevalence of concurrent physical and mental health conditions may be one of the mechanisms linking ageing among people who use drugs and the recent increase in DRDs (Scottish Drugs Forum, 2017).

Data from NDRDD suggest that women who died from DRD had poorer physical and mental health than their male counterparts, with a somewhat higher prevalence of physical and mental health conditions, long-term illness or disability, and recent acute hospital stays. Though the prevalence of concurrent physical health conditions increased among both men and women between 2009 and 2016, this increase appeared to be more marked among women (Information Services Division Scotland, 2018a). This is substantiated by figures from SDMD, which suggest that the prevalence of cooccurring health conditions among people attending treatment services for initial assessment has increased over the last decade and to a greater extent among women than men, particularly for mental health issues (Information Services Division Scotland, 2019).

The Older People with Drug Problems project in Scotland found that older women had substantially higher rates of hospitalisation for asthma/chronic obstructive pulmonary disease, lung cancer, and depression compared to their male peers, and compared to women in the rest of the population, whereas older men with drug problems tended to have higher rates of hepatitis C, liver disease, psychosis and heart disease (Scottish Drugs Forum, 2017). This suggests that comorbidity varies in complex ways with gender and with age, and points to some factors that may contribute to differential risk of drug-related death (for instance, poorer lung health among women).

These data should be interpreted with a number of caveats: for instance, women in the general population tend to have a higher prevalence of poor physical and mental health compared to men (Cheong et al., 2018; Scottish Public Health Observatory, 2020), and there are limited data from sources other than treatment services or death registrations. However, together they do suggest a greater increase in the prevalence of concurrent physical and mental health conditions among women who use drugs than their male peers, which may be contributing to increases in DRD.

In interviews with women who use drugs, many mentioned co-existing mental and physical health problems, which were often reported to be untreated or under-treated. These may have a direct impact on overdose risk, for instance, reduced lung function in the case of respiratory disease:

My partner, he’s saying to me, I think I’ve got that COPD [chronic obstructive pulmonary disease] or something, at night, it’s all bubbling and he said, he says it’s really scary, but, and I know I am, I get out of breath so easy, and it’s just wheezing…

Because I’m no getting any sleep, so it’s making me moody and it’s making me exhausted, moody, eh, [puff out of air], it’s starting to make my depression worse, because I’m nae [not] getting nae [any] blooming help because o’ the stigma, probably the doctors just looked at me and knowing I was an ex-addict, just looked and when ach, ken [know] what I mean, just gie [give] her that.

Poor mental health and a sense of hopelessness among women who use drugs was identified as an important factor by several professional stakeholders. This was felt to increase the risk of DRD, either directly by increasing the risk of deliberate (or ambivalent) overdose, or indirectly, by precipitating more risky consumption patterns. Several precipitating factors were described, including loss of access to children, bereavement, and changes to social security arrangements; interviewees also identified a reciprocal relationship between social isolation and mental health. The quotation below, in which a woman with lived experience describes her current mental state, illustrates a number of these themes:

Aye, sorry, I think it’s just one of them weeks, I used to sit for days and shut myself away for days, before I had the youngest two, but oh god that just gives [father of child] ammunition to take the bairns [children] off me, and oh god, I don’t want to lose the bairns again, so I was if I could just make it to the end of the day, and get to my bed, tomorrow will be a new day, but at times like now, I just think tomorrow is never coming, sorry.

Although NRS data indicate that deaths by suicide account for a greater percentage of DRD among women than men, the overall percentage of DRDs attributed to intentional self-poisoning among women is largely static or declining in recent years, reflecting a small increase in absolute numbers outstripped by a much greater rise in deaths attributable to accidental poisoning (supplementary Table S2.3). This suggests that the overall increase in DRD among women is unlikely to be driven by deaths by suicide, although it can be difficult to ascertain intentionality given that ambivalence of intention is common among people at risk of, or who have survived, overdose (Neale, 2000).

### Access to and engagement with treatment and harm reduction services

Both the total number and percentage of female clients undergoing initial assessment for specialist drug treatment have remained relatively stable over the past ten years in Scotland, with women accounting for a similar percentage of people identified from treatment settings compared to community-based surveys or figures on drug-related harms (supplementary Table S2).

Data from NDRDD found that between 2009 and 2016, a greater percentage of female than male decedents had:

- Been in contact with drug treatment services in the six months prior to death (53% vs 45%)
- Been prescribed opioid replacement therapy at the time of death (40% vs 27%)
- Experienced previous overdose (59% vs 49%)

Similarly, NESI found that women attending injecting equipment provision outlets were more likely to have been prescribed methadone in the last 6 months than men (82% vs 75% overall for the six sweeps between 2008/2009 and 2017/2018), indicating ongoing street drug use despite opioid replacement therapy .

These data suggest that women may be equally or more likely to access drug treatment services: the problem therefore may lie more with unmet needs or missed opportunities to intervene.

The interviews with women with lived experience identified several issues in this regard, including disagreements with providers about the appropriate dose and duration of opioid replacement therapy and a perceived lack of capacity and time, as illustrated below:

I’m only 20ml at [name], and they’ll no budge and put my methadone up, even though I went back onto heroin for a year, there’s still expecting me to get off the heroin, giving me 20ml of meth, which is impossible, you know, I just can’t do it.

Every time I asked them to drop me down, they wouldn’t drop me down, they kept telling me it was too early, but I knew I was ready to get dropped down, you know.

Conversely, some interviewees described positive experiences with services and support groups, though lack of awareness or misunderstandings about eligibility were cited as a barrier.

Professional stakeholders highlighted the potential impact of recent cuts to local drug treatment services, resulting in the closure of gender-sensitive services, higher thresholds for support, and reductions in staff pay, skill mix, and experience. Concerns were also raised by some stakeholders about reductions in provision of allied services which support people with drug problems, such as education, training, and employability. Lack of access to these, either due to service cuts or funding restrictions (e.g. lack of funds to cover travel expenses), were felt to hinder women’s efforts to achieve sustained reductions or cessation in drug use. This is consistent with recent reports by Public Health England and the Advisory Council on the Misuse of Drugs, which suggested that changes in drug treatment services in England may have been a contributory factor to rising DRD rates (Advisory Council on the Misuse of Drugs, 2016; Public Health England, 2017).

Some stakeholders raised the issue of gaps in harm reduction provision and potential unintended consequences of recovery-oriented approaches, such as a drive to reduce or cease opioid replacement therapy: this was also a theme identified in the Advisory Council on Misuse of Drugs and Public Health England reports (Advisory Council on the Misuse of Drugs, 2016). Some felt that women might be more vulnerable to the potential risks of a recovery-oriented treatment system, due to the greater salience of stigma and child protection concerns, and relational factors influenced by previous trauma.

With regard to harm reduction, the main source of information is naloxone supply and utilisation. Trends between 2008–2009 and 2017–2018 in the percentage of NESI survey participants reporting having been prescribed or carrying take-home naloxone (THN) were very similar by gender. As described in the subsequent section, naloxone uptake among female prisoners on release is relatively high. NDRDD analyses found that between 2009 and 2016, women who died were more likely than men have been in the same room as another individual at the time of death (42% vs 31%, among those for whom data were available), though rates of naloxone availability were similar, and the percentage of cases where available naloxone was administered was slightly higher among women (Information Services Division Scotland, 2018b). This suggests missed opportunities for naloxone use in overdoses occurring in both men and women, though there may be other variations in overdose response by gender that these data do not capture.

### Experiences of prison and re-settlement

Imprisonment was generally recognised by professional stakeholders as a difficult and disruptive event for women who use drugs, often accompanied by the loss of child custody, relationship breakdown, housing difficulties, and deteriorations in mental health. Problems experienced by women in the criminal justice system were mentioned by a number of stakeholders, including interruption to opioid replacement therapy on entry to prison and lack of throughcare support for issues like drug treatment, housing, and relationships.

Data from NDRDD shows that, during the period 2009–2016, female decedents were less likely than their male counterparts to have been in prison in the last 6 months (7% vs 17%) or ever in their lives (31% vs 57%), with no clear trend over time in either gender in the prevalence of prior imprisonment (Information Services Division Scotland, 2018b). This is largely consistent with the characteristics of the prison population in Scotland during this period (Scottish Government, 2020).

Prisons in Scotland appear to be performing relatively well with regard to women’s uptake of take-home naloxone at the point of release (Information Services Division Scotland, 2018c). However, whether this observation impacts on DRDs will depend on many other factors, including the likelihood of kits being carried and used, and of overdoses occurring in the presence of others.

### Economic and social trends, including austerity and welfare reform

There is increasing evidence that the health and social impacts of recent economic trends and austerity policies differ by gender (Engender, 2015; Glonti et al., 2015; Scottish Government, 2013; Thomson et al., 2018). For instance, women are more reliant on social security than men; are more likely to be lone parents or unpaid carers; and have borne a greater burden of recent cuts in social security provision and funding to public services (Engender, 2015; Scottish Government, 2013). Research from the Welfare Conditionality project has highlighted how the current social security system is poorly equipped to meet the needs of drug and/or mental health problems, with the increasing use of conditionality particularly harmful (Welfare Conditionality Project, 2018). Welfare reform and austerity have been identified as potential contributors to rising DRD rates in the UK in recent reports from national agencies (Advisory Council on the Misuse of Drugs, 2016; Public Health England, 2017).

This evidence was echoed by professional stakeholders, who highlighted that women who use drugs often experience an intersection of structural factors which may heighten vulnerability to the adverse impacts of austerity and welfare reform. Some were concerned that by reducing women’s income and financial autonomy, changes to the benefits system may have increased women’s vulnerability to abusive relationships, sexual exploitation, or commercial sex work, while one speculated that women are likely to be less assertive in accessing benefits they are entitled to, particularly in a context increasingly hostile to claimants. One service manager went as far as to state that poverty was the single biggest explanatory factor in the rise in DRDs in recent years.

Several of the women with lived experience interviewed had had benefits withheld or cut, largely as a result of missing appointments. In one case poor mental health was explicitly cited as the cause of loss of entitlements, reflecting challenges for this population in navigating the complexity of the social security system:

I’ve not been sanctioned, I was on DLA [Disability Living Allowance] due to my mental health, and because I’d relapsed and not picked up my medication, and not kept appointments, I never renewed the forms, so my benefits got cut in December, after them overpaying me for 7 months, and I’ve been told that I have to pay that back, and I don’t feel that’s my mistake, because I wasn’t made aware of it.

Another explanation raised by stakeholders was a hypothesized increase in homelessness among people who use drugs. However, data from NESI suggest no clear trend over time, overall or by gender, in the prevalence of homelessness among people attending injecting equipment provision outlets in Scotland (Health Protection Scotland & Glasgow Caledonian University, 2019). Similarly, data from NDRDD suggests no clear trend for either gender in the percentage of decedents who were homeless or living in temporary accommodation at the time of death (Information Services Division Scotland, 2018a). Although there appears to have been an increase in recent years in the percentage of decedents who had been in contact with homelessness services in the six months before death, this trend was similar by gender (Information Services Division Scotland, 2018a).

## Conclusions

**Figure 6:**
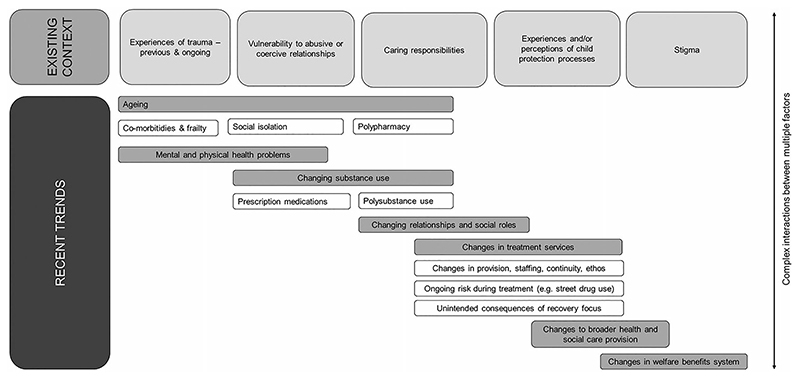
Schematic diagram summarising factors implicated in increasing rates of drug-related death among women in Scotland.

The question of what might explain the disproportionate rise in DRDs among women in Scotland is methodologically challenging, given the limitations of the data and the complexity of the phenomenon. Any explanation must involve multiple interacting factors. Figure 6 provides a simplified visual summary of our understanding to date, illustrating recent trends and their potential interaction with existing contextual factors which affect women’s experience of drug use.

This study has a number of strengths: it triangulates multiple sources (routine data, engagement with professional stakeholders, and interviews with women who use drugs), with a thematic matrix used to combine insights across different data sources in a systematic way. This enabled us to propose an integrated explanatory framework which can be applied and tested in future work. Its limitations reflect those of the included data sources, most of which rely heavily on people in contact with services or known to have experienced harms. These data sources also utilize the definition of DRD used in UK national statistics, which is designed to identify deaths associated with acute drug use and will exclude some deaths resulting from indirect or longer-term effects of drug use and other causes of mortality among people who use drugs (such as injecting-related infections or external causes such as accidents, assaults, and some suicides).

Future work to strengthen the evidence base in this area should focus on collecting or linking longitudinal data on rates and risk factors for drug-related and all-cause mortality among community-based cohorts of people who use drugs, with stratification by gender. This work could also include an investigation of the role of prescribable drugs. There is also a need for more in-depth qualitative research on gender in relation to drug-related death risk: in particular, the impact of welfare reform and public sector austerity, missed opportunities among people in treatment, and gendered aspects of naloxone supply and overdose response. Other areas identified for further research include an investigation of potentially ‘hidden’ populations of people who use drugs and the relationship between child protection proceedings and drug-related harms.

Although evaluating potential policy responses was not within the scope of the project, some implications can be drawn from this work. Our findings indicate important divergences in women’s experience of drug-related death risk, which suggest an important role for ‘gender mainstreaming’ for drug policy, practice, surveillance, and research (European Institute for Gender Equality, 2017). Such a response should identify where gender-sensitive approaches may be beneficial whilst recognising commonalities between genders and the diversity of experience and needs within them (Wincup, 2016), as well as addressing the intersections between gender and other forms of inequality, such as poverty (European Monitoring Centre for Drugs & Drug Addiction, 2017).

Our results also highlight the importance of involving women with lived experience in the design, delivery and evaluation of policy and services, recognizing that wider efforts to engage and empower people who use drugs have not always succeeded in reflecting the diversity of the population. Other implications identified by participants included the need for: more co-ordinated and holistic approaches across substance use treatment, mental health, physical health, and social support; trauma-informed and psychologically-informed services; child- and family-sensitive treatment services and support for family relationships; greater provision of employability, education, and volunteering opportunities; strategies to mitigate the adverse impacts of welfare reform; and efforts to address the universal problem of stigma and marginalization among people who use drugs. Assessing the impact and relative importance of these potential policy responses should be a priority for future participatory work involving people with lived experience, practitioners, policymakers, and researchers.

## Supplementary Material

Supplementary material

## Figures and Tables

**Table 1:** Routine data sources used in the study.

Name	Source of data	Responsible organisation	More information
Death registrations	National death registration data. As well as deaths classified by ICD codes as drug-related, all deaths where information on the death certificate is vague or suggests drugs may be implicated are followed up to ascertain whether they may be drug-related. A dedicated questionnaire is completed for each DRD by forensic pathologists.	National Records of Scotland (NRS)	https://www.nrscotland.gov.uk/statistics-and-data/statistics/statistics-by-theme/vital-events/deaths/drug-related-deaths-in-scotland
National Drug-Related Deaths Database (NDRDD)	Local DRD co-ordinators collect detailed information on the nature of DRDS and the health and social circumstances of individuals involved, using records from primary and secondary health care, drug treatment services, social work, police and prisons. The NDRDD is also routinely linked to data on hospital admissions and dispensed prescriptions.	Public Health Scotland (PHS); formerly Information Services Division (ISD)	http://www.isdscotland.org/Health-Topics/Drugs-and-Alcohol-Misuse/Drugs-Misuse/Drug-Related-Deaths-Database/
Scottish Drugs Misuse Database (SDMD)	Completion of a standard national dataset for people assessed by specialist drug treatment services^a^. Includes demographic information, prescribed and illicit drug profile, injecting risk behavior, social circumstances and contact with services.	Public Health Scotland (PHS); formerly Information Services Division (ISD)	http://www.isdscotland.org/Health-Topics/Drugs-and-Alcohol-Misuse/Drugs-Misuse/Scottish-Drug-Misuse-Database/
Drug-Related Hospital Statistics (DRHS)	Routine administrative data on hospital admissions associated with drug use from acute inpatient and day case hospital admissions (SMR01 records) and psychiatric inpatient and day case hospital admissions (SMR04 records).	Public Health Scotland (PHS); formerly Information Services Division (ISD)	http://www.isdscotland.org/Health-Topics/Drugs-and-Alcohol-Misuse/Drugs-Misuse/Drug-Related-Hospital-Statistics/
Needle Exchange Surveillance Initiative (NESI)	Cross-sectional biobehavioural survey of people who inject drugs with a focus on injecting risk behaviours and blood-borne virus prevalence. Participants recruited from selected agencies and pharmacies across Scotland which provide injecting equipment.	Health Protection Scotland/Glasgow Caledonian University	https://www.hps.scot.nhs.uk/a-to-z-of-topics/needle-exchange-surveillance-initiative-nesi/
Prevalence of problem drug use in Scotland	Successive studies undertaken every three years since 2000 have estimated the prevalence of problem drug use among those aged 15-64 years living in Scotland. For the purposes of the report, problem drug use is defined as the problematic use of opioids (including illicit and prescribed methadone use) and/or the illicit use of benzodiazepines, and implies routine and prolonged use as opposed to recreational and occasional drug use. Estimates are derived from capture-recapture methods using data from health services, specialist drug treatment, and the criminal justice sector.	Public Health Scotland (PHS); formerly Information Services Division (ISD)	The most recent report can be found at: https://www.isdscotland.org/Health-Topics/Drugs-and-Alcohol-Misuse/Publications/2019-03-05/2019-03-05-Drug-Prevalence-2015-16-Report.pdf

a ’Initial assessments’ in the Scottish Drugs Misuse Database (SDMD) refer to episodes of individuals first making contact with services providing tier 3 and 4 interventions (i.e. structured community or residential drug treatment) or reinitiating contact following a gap of at least six months since last attendance. Services contributing to the SDMD include specialist drug services and some medical services.
