# Supplementary material for: Why are drug-related deaths among women increasing in Scotland? A mixed-methods analysis of possible explanations
Source: Drugs (Abingdon Engl). Author manuscript; Available in PMC 2022 Jan 28. (PMC7612287; doi:10.1080/09687637.2020.1856786)
Supplement: Supplementary material [file EMS138310-supplement-Supplementary_material.pdf]

Supplementary material 1. Stakeholder summary.

	**Staff from drug and alcohol services, and the third sector**	
	Stakeholder description	Region of Scotland
1	Academic general practitioner	East
2	Service manager, drug and alcohol service	East
3	Team leader, addictions clinic	North
4	Senior clinical staff member, drug and alcohol service	West
5	Third sector professional (children and families focus)	East
6	GP and Allied Health Professional, homelessness service (2 participants)	East
7	Injecting equipment provision outlet staff – focus group (11 participants)	Various
8	Third sector advocacy organization staff – focus group (8 participants)	National remit
	**Analytical and academic staff**	
9	Analyst (local role)	
10	Analyst (national role)	
11	Analyst (national role)/academic (mixed methods)	
12	Clinical lead (national analytical role)	
13	Academic (primarily qualitative)	
14	Academic (primarily qualitative)	
15	Academic (quantitative)	
16	Academic (clinical)	
17	Group of academics: existing community of practice (primarily qualitative; 4 participants)	

Supplementary material 2. Additional tables and figures

Table S2.1. Comparison of numbers of drug-related deaths using the standard definition and consistent series, 2000-2018

	2000	2001	2002	2003	2004	2005	2006	2007	2008	2009	2010	2011	2012	2013	2014	2015	2016	2017	2018
Drug-related deaths: standard definition	292	332	382	317	356	336	421	455	574	545	485	584	581	527	614	706	868	934	1,187
of which:																			
Male	239	267	321	257	289	259	333	393	461	413	363	429	416	393	453	484	593	652	860
Female	53	66	61	62	67	77	87	62	113	132	122	155	165	134	161	222	275	282	327
Drug-related deaths: consistent series	293	339	388	330	365	346	430	474	590	570	512	606	604	557	621	707	871	935	1,187
of which:																			
Male	239	270	324	263	291	265	340	399	471	426	379	441	431	412	458	484	595	653	860
Female	54	70	64	69	74	81	89	75	119	144	133	165	173	145	163	223	276	282	327
'Extra' deaths counted in consistent series	1	7	6	13	9	10	9	19	16	25	27	22	23	30	7	1	3	1	0
of which:																			
Male	0	3	3	6	2	6	7	6	10	13	16	12	15	19	5	0	2	1	0
Female	1	4	3	7	7	4	2	13	6	12	11	10	8	11	2	1	1	0	0

Percentage increase in annual average number of deaths between 2004-08 and 2014-18

Standard definition	
Male	75
Female	212
Consistent series	
Male	73
Female	190

Source: National Records of Scotland. Broadly speaking, the standard definition counts deaths on the basis of the drugs at the time of death, whereas the consistent series counts death on the basis of the classification of the drugs at the end of the latest year which is covered by the publication (in this case 2018). See (National Records of Scotland, 2018) for more detail.

Table S2.2. Evidence from existing data sources regarding the size and demographic characteristics of the population of people who use drugs in Scotland

				Trend over time		
**Data source**	**Population**	**Indicator**	**Period of time**	**Gender profile**	**Recruitment into drug use**	**Age/gender profile**
Scottish Crime and Justice Survey (SCJS)	Representative sample of people living in private households	Self-reported drug use	2014/15 – 2017/18	Slight increase in prevalence of any drug use among women in most recent sweep, following a longer-term static/declining trend; however, prevalence of past-year use of drugs most commonly implicated in DRD (heroin, methadone, tranquilisers) among women is largely stable between 2014/15 and 2017/18	n/a	Not available
Scottish Drug Misuse Database (SDMD)	People undergoing initial assessment for specialist drug treatment	Demand for/access to specialist drug treatment, and demographic characteristics of treatment population	2006/07 – 2017/18	Largely stable over time, ranging between 28-31% female	Increasing median time since self-reported onset of injecting among both genders – trend similar	Proportion of clients aged ≥35 years greater among men than women, but grew more steeply among women Trend in median age largely similar between genders, with slight narrowing of age gap between men and women
Needle Exchange Surveillance Initiative (NESI)	Attendees of injecting equipment provision outlets	Demographic characteristics of people who inject drugs	2008/09 to 2017/18	Largely stable over time, ranging between 27-30% female	Increasing average time since self-reported onset of injecting among both genders – trend similar	Increasing median age over time with similar trend in both genders
Estimates of prevalence of problem drug use in Scotland	People with regular problematic use of opioids or benzodiazepines, identified by capture-recapture modelling using health care, social work, police, and prison records	Number and demographic characteristics of people with problem drug use	2003; 2006; 2009/10; 2012/13; 2015/16<br>N.B. methodological changes mean comparisons should be treated with caution	Largely stable over time:<br>2003: 31% female (n=15,793)<br>2006: 30% female (n=16,598)<br>2009/10: 29% female (n=17,300)<br>2012/13: 30% female (n=18,200)<br>2015/16: 29% female (n=16,600)	n/a	n/a
Drug-related hospital stays (DRHS)	Inpatient and day case activity attributable to drugs (includes both mental & behavioural disorders and poisoning/overdose)	Drug-related harms requiring inpatient or day case hospital care	1996/97 – 2017/18	Increasing numbers of stays among both men and women but trend much steeper among men since 2012/13	When restricting to new patients*, trends similar to all stays – increasing number among both genders with steeper trend among men since 2012/13	Increasing median age over time with similar trend in both genders

*A new patient is defined as an individual admitted to hospital as an inpatient or day case patient within a given time period (e.g. financial year),who has not had a similar drug-related stay in hospital within the previous ten years.

Table S2.3. Number of drug-related deaths between 2011 and 2018 by underlying cause of death and gender, using the NRS ‘new’ coding rules as applied from 2011 onwards.

	Year	Cause of death category (ICD-10 code)											
		Drug abuse (F11-F16, F19)		Accidental poisoning (X40-X44)		Intentional self-poisoning (X60-X64)		Assault by drugs, etc (X85)		Undetermined intent (Y10-Y14)		All	
		n	%	n	%	n	%	n	%	n	%	n	%
Female													
	2011	3	1.9	77	49.7	16	10.3	0	0.0	59	38.1	155	100.0
	2012	7	4.2	85	51.5	34	20.6	0	0.0	39	23.6	165	100.0
	2013	3	2.2	88	65.7	17	12.7	0	0.0	26	19.4	134	100.0
	2014	8	5.0	111	68.9	20	12.4	0	0.0	22	13.7	161	100.0
	2015	16	7.2	160	72.1	23	10.4	0	0.0	23	10.4	222	100.0
	2016	9	3.3	213	77.5	30	10.9	0	0.0	23	8.4	275	100.0
	2017	8	2.8	236	83.7	23	8.2	0	0.0	15	5.3	282	100.0
	2018	9	2.4	273	83.5	26	8.0	0	0.0	20	6.1	327	100.0
Male													
	2011	9	2.1	269	62.7	20	4.7	0	0.0	131	30.5	429	100.0
	2012	19	4.6	280	67.3	31	7.5	0	0.0	86	20.7	416	100.0
	2013	19	4.8	278	70.7	33	8.4	1	0.3	62	15.8	393	100.0
	2014	24	5.3	360	79.5	25	5.5	0	0.0	44	9.7	453	100.0
	2015	33	6.8	393	81.2	31	6.4	0	0.0	27	5.6	484	100.0
	2016	23	3.9	517	87.2	18	3.0	0	0.0	35	5.9	593	100.0
	2017	26	4.0	571	87.6	31	4.8	0	0.0	24	3.7	652	100.0
	2018	37	4.3	744	86.5	33	3.8	0	0.0	46	5.3	860	100.0

Source: National Records for Scotland (bespoke request). For more information on coding rules applied to the underlying cause of death, see (National Records of Scotland, 2017a).

Figure S2.1. Percentage of deaths in which only one drug (and perhaps, alcohol) was implicated, by gender and year

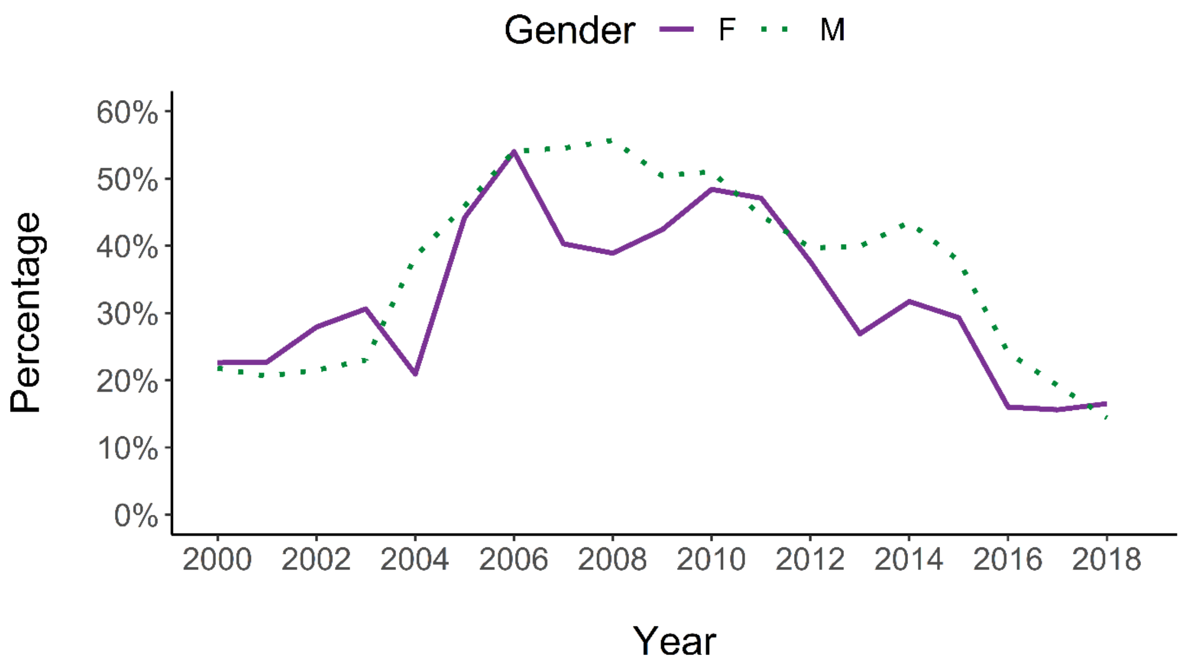

Source: National Records of Scotland

Figure S2.2a. Percentage of DRDs where the deceased was a parent or parental figure to children under 16 years, by gender and year.

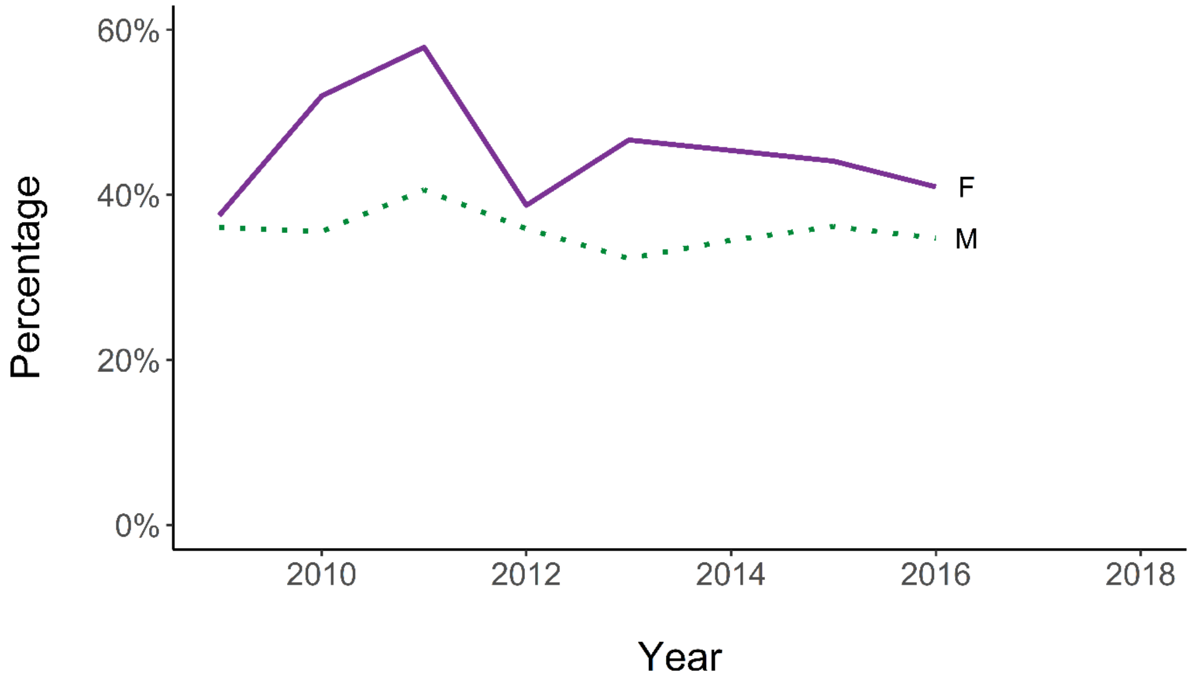

Source: National Drug-Related Deaths Database, Public Health Scotland

Figure S2.2b. Percentage of DRDs where the deceased was living with children under 16 years at the time of death, by gender and year.

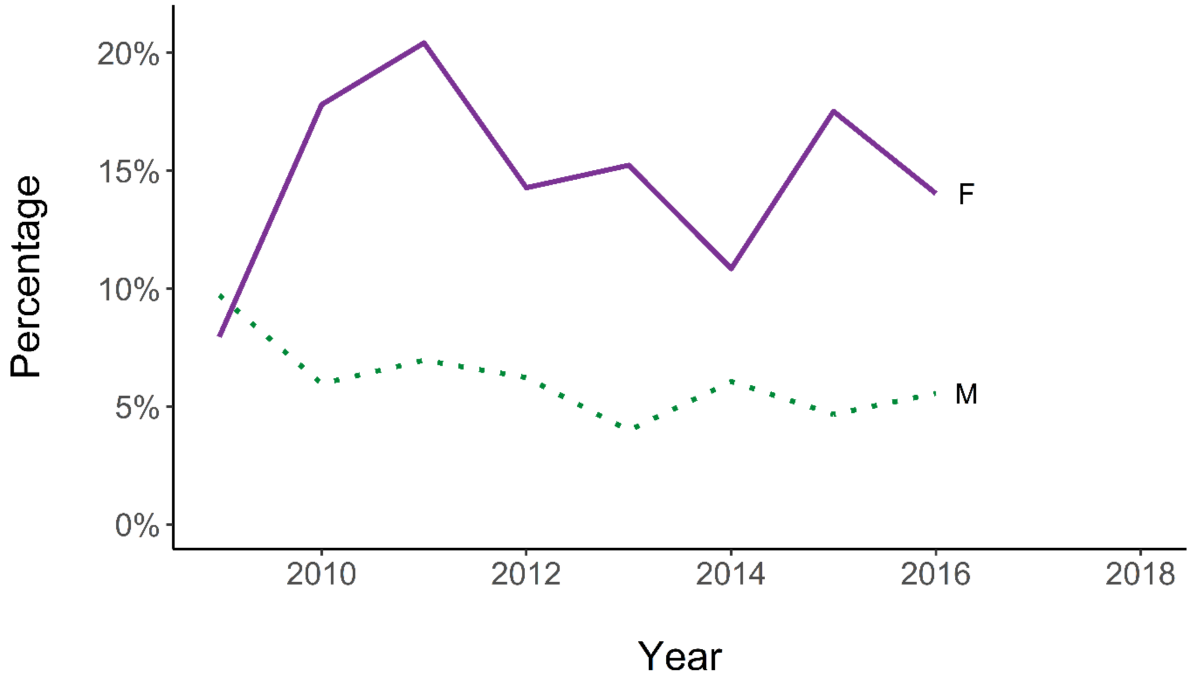

Source: National Drug-Related Deaths Database, Public Health Scotland
